# Neuropathological features of genetically confirmed DYT1 dystonia: investigating disease-specific inclusions

**DOI:** 10.1186/s40478-014-0159-x

**Published:** 2014-11-18

**Authors:** Reema Paudel, Aoife Kiely, Abi Li, Tammaryn Lashley, Rina Bandopadhyay, John Hardy, Hyder A Jinnah, Kailash Bhatia, Henry Houlden, Janice L Holton

**Affiliations:** Department of Molecular Neuroscience, UCL Institute of Neurology, London, UK; Queen Square Brain Bank, UCL Institute of Neurology, London, UK; Department of Neurology, Human Genetics, and Paediatrics, Emory University, Atlanta, Georgia 30322 USA

**Keywords:** DYT1, Neuropathology, Isolated dystonia, Inclusions

## Abstract

**Introduction:**

Early onset isolated dystonia (DYT1) is linked to a three base pair deletion (ΔGAG) mutation in the *TOR1A* gene. Clinical manifestation includes intermittent muscle contraction leading to twisting movements or abnormal postures. Neuropathological studies on DYT1 cases are limited, most showing no significant abnormalities. In one study, brainstem intraneuronal inclusions immunoreactive for ubiquitin, torsinA and lamin A/C were described. Using the largest series reported to date comprising 7 DYT1 cases, we aimed to identify consistent neuropathological features in the disease and determine whether we would find the same intraneuronal inclusions as previously reported.

**Result:**

The pathological changes of brainstem inclusions reported in DYT1 dystonia were not replicated in our case series. Other anatomical regions implicated in dystonia showed no disease-specific pathological intracellular inclusions or evidence of more than mild neuronal loss.

**Conclusion:**

Our findings suggest that the intracellular inclusions described previously in DYT1 dystonia may not be a hallmark feature of the disorder. In isolated dystonia, DYT1 in particular, biochemical changes may be more relevant than the morphological changes.

**Electronic supplementary material:**

The online version of this article (doi:10.1186/s40478-014-0159-x) contains supplementary material, which is available to authorized users.

## Introduction

Dystonia is a common and heterogeneous neurological disorder. Recently, dystonia has been defined as a movement disorder characterized by sustained or intermittent muscle contractions causing abnormal, often repetitive, movements, postures, or both with genetic heterogeneity reported. The term ‘dystonia musculorum deformans’ was proposed in 1911 to describe a disorder in children with twisted postures, muscle spasms, gait abnormalities and clonic, rhythmic jerking movements [1]. In childhood onset cases dystonia tends to become generalized, while remaining focal or segmental in adult onset cases [2]. A newly proposed classification system classified dystonia according to two axes: clinical characteristics and etiology [3]. Dystonia linked to a 3 base pair deletion (ΔGAG) mutation in exon 5 of the *TOR1A* gene on chromosome 9q34.11 is called DYT1 dystonia [4]. The new classification system classified DYT1 dystonia as isolated dystonia in which dystonia is the only clinical sign with the exception of tremor [3]. The prevalence of isolated dystonia was estimated at 330 per million in Rochester, Minnesota [5] and is about 152 per million in the European population [6]. In DYT1 the inheritance is autosomal dominant with the disease onset in childhood [7] and the most common clinical features include muscle contractions affecting the leg or arm, often progressing to generalized involvement with severe disability [8]. The mutation has been identified in many diverse ethnic groups [9,10] and has a low penetrance of 30–40% [10-12].

The *TOR1A* gene encodes a 332 amino acid protein, torsinA, which has a cytoplasmic localization in cells. TorsinA is widely expressed throughout the central nervous system in humans, but is found at particularly high levels in dopaminergic neurons of the substantia nigra, locus coeruleus, Purkinje cells, cerebellar dentate nucleus, basis pontis, thalamus, hippocampal formation, oculomotor nuclei and frontal cortex [13-16]. The exact function of torsinA remains unclear in humans. However, mutant torsinA protein has been shown to have aberrant cellular localization and impaired protein interactions and is associated with defective synaptic vesicle formation and altered development of neuronal pathways [17].

Much of the research in primary dystonia has focused on the role of the basal ganglia in disease [18-20]. However, imaging studies of patients carrying a mutation in *TOR1A* causing primary dystonia revealed an increase in metabolic brain activity not only in the basal ganglia but also in the cerebellum [21,22]. Neuropathological investigation of clinically diagnosed primary dystonia cases has been generally disappointing with no specific abnormalities observed [23]. In genetically confirmed DYT1 cases, no evidence of neuronal loss, inflammation or altered localization of torsinA could be identified [15,23-26]. A possible reduction in striatal dopamine and the size of pigmented neurons in the substantia nigra have been suggested [15,23,26]. The most interesting observation to date has been the finding of perinuclear intraneuronal inclusions immunoreactive for ubiquitin, torsinA and lamin A/C in the periaqueductal grey matter, cuneiform and pedunculopontine nuclei [27]. Similar inclusions were reported in some of the DYT1 mouse models produced by expression of transgenic human torsinA but this has not been a consistent finding [28,29]. The intriguing observation of brainstem inclusions in DYT1 cases has not, so far, been replicated.

The aims of this current study were: 1) to report the neuropathological features of seven previously unreported genetically proven DYT1 cases, 2) to determine whether any pathological features were consistently observed in all cases and could be regarded as disease-related and 3) to determine whether the perinuclear intracytoplasmic inclusions previously described in DYT1 dystonia are a consistent feature of the disease.

## Materials and methods

### Cases

This project was approved by the Joint Local Research Ethics Committee of the National Hospital for Neurology and Neurosurgery and the UCL Institute of Neurology. Autopsy specimens of 7 DYT1 confirmed cases were received from the Brain and Tissue Banks for Developmental Disorders, Baltimore. Clinical details, macroscopic findings and neuropathology reports were provided by the institution. To our knowledge, none of the DYT1 cases have been previously reported. Formalin fixed, paraffin embedded brain tissue was available in all dystonia cases although systematic sampling of multiple regions for each case was not possible due to the retrospective nature of the study. Where possible the frontal and temporal cortices, striatum, globus pallidus, thalamus, subthalamic nucleus, cerebellum, midbrain, pons, and medulla were investigated. Brain regions available for study in each case are indicated in Additional file 1: Table S1. The midbrain was absent in cases 2 and 3 and was only partially represented in case 6. The pons including the reticular formation was present in all cases except case 6.

### Genetics

DNA was extracted from frozen brain tissue (cerebellum or frontal cortex) using DNeasy Blood and Tissue Kit (Cat no 69504). Cases were sequenced for the recurring ΔGAG mutation in exon 5 of *TOR1A* using standard Sanger polymerase chain reaction sequencing on an ABI 3730XL machine (Applied Biosystems, Inc., Foster City, CA, USA) and analyzed using Sequencher software.

### Immunohistochemistry (IHC)

In brief, paraffin embedded tissue sections (7 μm) were cut and stained using routine methods, including hematoxylin and eosin, Gallyas silver and Luxol fast blue/cresyl violet. IHC staining was performed according to a standard avidin-biotin complex protocol using the following antibodies: ubiquitin (1:200, Dako, Ely,UK), tau (1:600, AT8, Autogen Bioclear, Calne, UK); AT100 (1:200, Innogenetics, Gent, Belgium), glial fibrillary acidic protein (GFAP; 1:1,000, Dako), Aβ (1:200, Dako), α-synuclein (1:50, Novacastra, Newcastle, UK), α-synuclein (1:1000, BD Transduction Biolabs, Oxford, UK), p62 (1:100, BD Bioscience, Oxford, UK), CD68 (1:150, Dako), and TDP-43 (1:2000, Protein Tech, Manchester, UK). Antibodies to ubiquitin, p62, and tau required pretreatment by pressure cooking in sodium citrate buffer, pH 6.0, for 10 minutes before IHC staining. Sections to be used for IHC staining with antibodies to α-synuclein and Aβ were treated with formic acid for 30 minutes before pressure cooking as described above. For GFAP IHC, pre-treatment with proteinase K was used. Antibody binding sites were visualized using the chromogen diaminobenzidine, and sections were counterstained using Mayer’s hematoxylin.

### Neuropathology assessment

The staining protocol was designed to detect intracellular inclusions as previously described in DYT1 dystonia [27]. In all cases brain regions including the midbrain and pons were stained with antibodies to ubiquitin and p62 to assess the presence of intraneuronal inclusions [27]. Due to limited tissue availability the periaqueductal grey matter was not available in all cases (absent in cases 2, 3 and 6). The pontine tegmentum was available in all cases except case 6. Cases were systematically screened for additional neuropathological changes. Using Aβ IHC vascular and parenchymal Aβ deposition was determined in the frontal cortex and diffuse and mature plaque load was assessed based on the criteria of the Consortium to Establish a Registry for Alzheimer’s Disease (CERAD) [30]. Neurofibrillary tangle (NFT) pathology was determined using tau IHC and staged where possible according to the method of Braak and Braak [31]. Vascular pathology was assessed and tau, ubiquitin, and α-synuclein IHC were performed in selected regions to exclude progressive supranuclear palsy, corticobasal degeneration, Parkinson disease and multiple system atrophy as described previously [32]. Where brainstem Lewy bodies (LBs) were identified cortical LB pathology was also determined [33]*.*

## Results

### Genetics

Sanger sequencing confirmed that all the cases harbour the ΔGAG mutation (p.303-/Glu304) in exon 5 of the *TOR1A* gene which is a recurring mutation in DYT1 dystonia.

### Clinical data

The demographic and clinical data for the cases are summarized in Table 1.

**Table 1:** **Demographics and clinical details of DYT1 cases**

***Case***	***Sex***	***Ethnicity***	***Phenotype***	***Family history***	***Age at onset, years***	***Age at death, years***	***Cause of death***
1	F	American Caucasian	Non-manifesting	+	N/A	89	Natural
2	F	American Caucasian	Non-manifesting	+	N/A	87	Stroke
3	F	American Caucasian	Affected	-	~9-10	77	Complications of disorder
4	F	American Caucasian	Non-manifesting	+	N/A	90	Stroke
5	F	American Caucasian	Affected	+	N/A	90	Natural
6	F	American Caucasian	Non-manifesting	N/A	N/A	88	N/A
7	M	American Caucasian	Non-manifesting	N/A	N/A	75	Natural

N/A: not available.

Case 1: This Caucasian female (ΔGAG mutation carrier) was 89 years old at death. Despite having a strong family history of dystonia she was clinically unaffected. Her grandson had torticollis. Her sister (case 2) and niece were positive for the ΔGAG mutation. She had a medical history of hypertension, coronary artery disease, arterial vascular insufficiency involving left arm and developed cognitive impairment.

Case 2: A Caucasian woman (ΔGAG mutation carrier) who died at the age of 87 years had a past medical history of hypertension, hyperlipidemia, coronary artery disease, pulmonary embolism, hypothyroidism, osteoarthritis and dementia. Although unaffected herself, she had a family history of dystonia. Her sister (case 1) and niece were both positive for ΔGAG mutation. She died following a right middle cerebral artery infarction confirmed prior to death by imaging studies.

Case 3: This Caucasian female, positive for ΔGAG mutation, had a clinical diagnosis of dystonia with life-long disability. Dystonic symptoms started at the age of 9 or 10 and were slowly progressive. She had a similarly affected sibling but no other family history was available. By 73 years of age she was weak bilaterally in the upper and lower limbs and wheelchair bound. Abnormal involuntary movements affected both upper extremities. She complained of progressive bilateral hearing loss and episodic problems with her vision and speech. Neurological examination reported no facial asymmetry, weakness in the both upper and both lower extremities, more severe proximally than distally. Deep tendon reflexes were diminished in the upper extremities, but symmetrical. Sensory evaluation showed intact pinprick and vibration sensation in both hands and right foot but these were absent in the left leg. No obvious cerebellar dysfunction was reported. She died at the age of 77.

Case 4: A Caucasian female (ΔGAG mutation carrier) of Jewish ancestry died at the age of 90 years. She had been well until the age of 89 when she developed left sided weakness, expressive aphasia and dysphagia secondary to stroke. She also had intermittent confusion and paranoia. MRI reportedly showed an infarct involving the right internal capsule, thalamus and white matter. She did not have a history of movement disorder; however, her grandson had a diagnosis of dystonia.

Case 5: A Caucasian female with a clinical diagnosis of dystonia died at the age of 90 years. Her son had a confirmed ΔGAG mutation. No further medical history was available.

Case 6: This Caucasian female was a ΔGAG mutation carrier and died aged 88 years. No further medical history was available.

Case 7: This Caucasian male (ΔGAG mutation carrier) was unaffected by dystonia and died at the age of 75. No further clinical history was available.

### Neuropathology assessment

A summary of the pathological findings is given in Table 2.

**Table 2:** **Summary of pathological findings in DYT1 cases**

***Case***	Brain weight (g)*	***Infarction***	***Aβ pathology***		***Tau pathology***	***Lewy body pathology***	***SVD***	***CAA***	Final neuropathological diagnosis*
			***D***	***M***					
1	N/A	-	+	-	-	-	+	-	• moderate atherosclerosis of cerebral vessels
									• mild neuronal loss and gliosis in hippocampus between the CA1 region and the subiculum
									• mild loss of Purkinje cells in the cerebellum
									• mineralization of vessel walls in the dentate nucleus
									• non-specific neurodegenerative changes
2	1110	globus pallidus	+	+	-	-	+++	+	• mild cerebral artery atherosclerosis
									• right middle cerebral artery territory subacute infarct
									• possible AD
3	1200	-	-	-	-	-	-	-	• mild cortical atrophy
									• small areas of infarction
									• cerebral athero-arterio-arteriolosclerosis, remote frontal white matter infarct
4	1010	globus pallidus	+++	++	+ (cortex, striatum and brainstem)	-	+	++	• cerebral artho-arteriosclerosis (a) microinfarcts in cerebral white matter, deep gray nuclei and occipital cortex (b) macro infarct in right posterior internal capsule and pulvinar
									• hippocampal sclerosis
5	N/A	midbrain tegmentum	++	++	+ (cortex, striatum and brainstem)	++ (cortical and brainstem)	+	-	• diffuse LB disease (a) substantia nigra degeneration with LBs (b) neocortical LBs (c) AD neuropathology
									• cerebrovascular disease (a)numerous infartcs of different ages (b) atherosclerotic disease of circle of Willis arteries
6	1184	-	++	+	+ (cortex, striatum and brainstem)	-	+	+	• brain generalized atrophy
									• focal acute (perimortem) petechial hemorrhages
7	N/A	-	-	-	+ (brainstem)	+ (substantia nigra)	-	-	• melanocytic neoplasm with diffuse leptomeningeal and perineural spread

- = absent; + = occasional/mild; ++ = moderate; +++ = severe; N/A-not available; SVD- Small vessel disease; CAA- Cerebral amyloid angiopathy; Aβ: D-diffuse plaques, M-mature plaques; AD: Alzheimer’s disease; LB: Lewy body; *: Derived from neuropathology report issued by BTDBB.

#### Macroscopic findings

The macroscopic details were available from the neuropathology reports supplied by the brain bank providing the case. In all the DYT1 cases the weight of the unfixed brains was in the normal range [34] or mildly reduced (range 1010 – 1200 g, data available for cases 2–4 and 6). The blood vessels of the circle of Willis showed variable atherosclerosis in all cases except case 7. Mild cerebral cortical atrophy was evident in case 3 and generalized brain atrophy consistent with age was described in case 6. At brain slicing the cortical ribbon was well preserved in all cases. The ventricular system was unremarkable in cases 3 and 4 but there was moderate dilatation in case 2. Four cases showed cerebral infarction, this affected the right middle cerebral artery territory in case 2, frontal white matter in case 3, several infarcts of varying age with unspecified distribution in case 5, and multiple regions of cerebral white matter, occipital cortex and deep grey nuclei in case 4. Case 7 was found to have a diffuse leptomeningeal melanocytoma. Pigmentation of the substantia nigra and locus coeruleus was described in cases 2, 3, 4, and 6 where it was noted to be normal.

#### Histological findings

Examination of the midbrain and pons, represented in all cases except cases 6, using ubiquitin and p62 IHC showed no evidence of NCIs of similar distribution and morphology to those previously associated with early-onset DYT1 dystonia [27]. Moreover a detailed examination of additional brain regions did not reveal similar inclusions in other structures.

DYT1 cases were systematically assessed for other neuropathological abnormalities. Neuronal loss was generally inconspicuous in the regions studied. There was mild neuronal cell loss in the pigmented neurons of the substantia nigra pars compacta of case 1, 4, 5 and 7 and in the locus coeruleus of case 2, 4 and 5. Mild to moderate Purkinje cell loss was observed in the cerebellum of all the cases. Mild to moderate gliosis of the striatum was observed in all the cases and also in the substantia nigra of cases 1, 4 5, 6 and 7 in which it was available for analysis. The pontine base showed variable gliosis and this was severe in case 3. Vascular pathology and ischemic damage in the form of small vessel disease (SVD), cerebral amyloid angiopathy (CAA) and infarction was assessed in all cases. Cases 1, 4, 5 and 6 had mild SVD while this was severe in case 2. CAA was mild in cases 2 and 6 and moderate in case 4. Small areas of infarction were observed in 3 cases. In cases 2 and 4 infarcts involved the globus pallidus and in case 5 a small infarct was noted in the midbrain tegmentum. We used two different α-synuclein antibodies for IHC in multiple regions to assess LB pathology. LBs were observed in case 5 and rare LBs were observed in the substantia nigra of case 7. Sparse diffuse Aβ plaques were noted in the cortex of case 1. In cases 2, 4, 5 and 6 both diffuse and mature cortical Aβ plaques were present. Tau pathology in the form of NFTs and neuropil threads was evident in four cases (cases 4, 5, 6 and 7).

No consistent abnormalities were noted in the brain regions available for examination in this study including the midbrain, striatum, globus pallidus, cerebellum, and pons. Apart from limited Alzheimer’s and LB pathology as described above, none of the cases were found to have neuropathological features of any other neurodegenerative disease. In particular, multiple system atrophy, progressive supranuclear palsy and corticobasal degeneration were excluded.

Additional file 1: Table S1 summarizes the semi-quantitative findings of the histological features of the seven DYT1 cases.

## Discussion

There are only five neuropathological studies of genetically confirmed DYT1 mutation cases in the literature [23]. We aimed to significantly contribute to the field of dystonia research by assessing the largest case series of DYT1 yet investigated to determine whether consistent neuropathological features could be identified as a hallmark of the disease. This study also addressed the question of whether neuronal perinuclear intracytoplasmic inclusions of the type previously associated with early-onset DYT1 dystonia could be confirmed in our cases [27].

We confirmed that all of the cases carry a 3-basepair deletion (ΔGAG) in exon 5 of the *TOR1A* gene which is a common mutation in DYT1 dystonia. Among the seven cases studied, two cases (cases 3 and 5) were symptomatic and five were asymptomatic carriers for DYT1 dystonia (cases 1, 2, 4, 6, and 7). Using a similar IHC approach to that applied in a previous neuropathological study of DYT1 dystonia [27] we did not replicate the finding of ubiquitin positive inclusions in the midbrain and pons with the exception of inclusions that were also immunoreactive for tau or α-synuclein, representing NFTs and LBs respectively. We also screened for intracellular inclusions in other brain regions and could not demonstrate abnormality other than sparse Alzheimer and LB pathology. The remaining pathological features identified in the cases were those of gliosis, mild-moderate Purkinje cell depletion, CAA, SVD of varying severity and cerebral infarcts. None of these changes can be regarded as disease specific and are most likely to be due to the advanced age at death of the cases (75–90 years).

We examined anatomical regions implicated in dystonia and found no pathological intracellular inclusions or evidence of more than mild neuronal loss in the caudate, putamen, subthalamic nucleus, thalamus, pigmented neurons of the substantia nigra and globus pallidus, other than that associated with small infarcts in the latter in two cases [35].

The reason that we failed to replicate the findings of McNaught *et al.* [27] is uncertain. With the exception of case 4 in the McNaught series, who was aged 33 years at death, the ages of our 2 case series were similar excluding the possibility that the inclusions observed are age related. One difference between the two studies is that all four cases described by McNaught *et al.* manifested clinical dystonia compared with only two cases in our series, the remaining five cases that we investigated were non-manifesting carriers of the gene mutation. If the neuronal inclusions are only present in patients with clinical dystonia this may have reduced the likelihood that we would have detected inclusions in our cases. However, similar electrophysiological and functional imaging abnormalities have been reported in cortical motor circuits of manifesting and non-manifesting DYT1 carriers [36,37]. An alternative explanation could be that different antibodies were used in the two studies and this remains a possibility. Given the limitation that only a small amount of tissue was available for study we chose ubiquitin and p62 as screening antibodies as they target abnormally folded proteins for degradation by the proteasome and autophagy respectively. As both are sensitive markers of inclusions formed from a wide variety of different proteins in neurodegenerative diseases and can recognise proteinaceous inclusions for which the principle protein component has not been identified we argued that if DYT1 dystonia is associated with intraneuronal inclusions these would be highlighted by one or both of these antibodies [38,39]. Any inclusions identified could then have been further defined using other specific antibodies such as lamin A/C or torsinA and the affected neurons would have been immunophenotyped to determine whether cholinergic neurons were affected as described in the McNaught study. A limitation of our study was that, due to the restricted tissue available for examination, not all regions were present in each case. It therefore remains a possibility that because of these sampling issues we did not identify rare inclusions. Future studies in systematically sampled cases would be required to resolve this issue. Assessment of cell inclusions using a reliable human specific torsinA antibody would be highly desirable. We have previously tested 4 different antibodies: torsinA [13,27,40], torsinA (TA913) [41], anti-torsinA (ab34540) and torsinA (Santacruz s-20) using western blots, IHC and immunofluorescence (unpublished data). However, none of these was found to be reliable and therefore could not be included in the study.

Despite the limitations of this study due to its retrospective nature and the restricted tissue availability our observations support the previous findings that in primary dystonia, there is no overt neurodegeneration or cell loss [23]. The pathological change of brainstem inclusions reported in DYT1 dystonia [27] was not replicated in our case series. Hence this study supports the previous findings that overt cell loss or other obvious neuropathological defects such as inclusions do not seem to be consistently observed in DYT1 dystonia. However, subtle morphological defects of the type seen in the DYT1 mouse models (e.g. abnormalities of neuronal size/shape, dendrite structure, spines, etc.) cannot be excluded. The analogy is similar to most human mental retardation syndromes, where cell loss or inclusions are absent, but marked abnormalities of neuritic structure are thought to play an important role. The subtle morphological changes may not be identifiable using current methods. In this regard, generation of cellular disease models using the induction of pluripotent stem (iPS) cells from patient skin fibroblast has opened new horizons [42,43]. Understanding the cellular pathways influenced by particular mutations and the neuronal networks affected may inform future neuropathological studies.

## Conclusion

We report a detailed neuropathological study of 7 DYT1 cases, the largest DYT1 case series yet investigated. The anatomical regions implicated in dystonia showed no consistent disease specific pathological features. Our observations support the previous findings that understanding the biochemical changes may be more relevant than morphological alterations in isolated dystonia.

## Additional file

Additional file 1: Table S1. Semi-quantitative summary of neuronal loss (NL), gliosis pathology, Lewy bodies (LB), Tau pathology and Cerebral amyloid angiopathy (CAA) in DYT1 cases.

## Abbreviations

NCIs: Neuronal cytoplasmic inclusions
CERAD: Consortium to establish a registry for alzheimer’s disease
NFT: Neurofibrillary tangle
IHC: Immunohistochemistry
GFAP: Glial fibrillary acidic protein
LB: Lewy body
SVD: Small vessel disease
CAA: Cerebral amyloid angiopathy
