# Supplementary material for: Neuropathological features of genetically confirmed DYT1 dystonia: investigating disease-specific inclusions
Source: Acta Neuropathol Commun. 2014 Nov 18;2:159. doi: 10.1186/s40478-014-0159-x (PMC4247124; doi:10.1186/s40478-014-0159-x)
Supplement: Additional file 1: Table S1. — Semi-quantitative summary of neuronal loss (NL), gliosis pathology, Lewy bodies (LB), Tau pathology and Cerebral amyloid angiopathy (CAA) in DYT1 cases. [file 40478_2014_159_MOESM1_ESM.docx]

| **Additional file 1: Table S1: Semi-quantitative summary of neuronal loss (NL), gliosis pathology, Lewy bodies (LB), Tau pathology and Cerebral amyloid angiopathy (CAA) in DYT1 cases** | | | | | | | | | | | | | | | | | | | | | | | | |
| --- | --- | --- | --- | --- | --- | --- | --- | --- | --- | --- | --- | --- | --- | --- | --- | --- | --- | --- | --- | --- | --- | --- | --- | --- |
| Brain regions | Case 1 | | | | | | Case 2 | | | | | | Case 3 | | | | | | Case 4 | | | | | |
|  | NL | Gliosis | Aβ | | LB | Tau | NL | Gliosis | Aβ | | LB | Tau | NL | Gliosis | Aβ | | LB | Tau | NL | Gliosis | Aβ | | LB | Tau |
|  |  |  | D | M |  |  |  |  | D | M |  |  |  |  | D | M |  |  |  |  | D | M |  |  |
| Frontal cortex | - | + | + | - | - | - | - | +++ | + | + | - | - | - | ++ | - | - | - | - | - | ++ | ++ | + | - | + |
| Temporal cortex | - | + | - | - | - | - | - | - | + | + | - | - | - | - | - | - | - | - | - | ++ | ++ | + | - | - |
| Caudate | - | + | N/P | | - | - | - | ++ | N/P | | - | - | - | + | N/P | | - | - | - | ++ | N/P | | - | + |
| Putamen | - | + | N/P | | - | - | - | ++ | N/P | | - | - | - | ++ | N/P | | - | - | - | ++ | N/P | | - | ++ |
| Globus pallidus | - | ++ | N/P | | - | - | - | +++ | N/P | | - | - | N/A | N/A | N/A | | N/A | N/A | - | +++ | N/P | | - | - |
| Thalamus | - | + | N/P | | - | - | - | - | N/P | | - | - | N/A | N/A | N/A | | N/A | N/A | - | ++ | N/P | | - | - |
| Subthalamic nucleus | - | + | N/P | | - | - | N/A | N/A | N/A | | N/A | N/A | N/A | N/A | N/A | | N/A | N/A | N/A | N/A | N/A | | N/A | N/A |
| Midbrain tegmentum | - | ++ | N/P | | - | N/P | N/A | N/A | N/A | | N/A | N/A | N/A | N/A | N/A | | N/A | N/A | - | - | N/P | | - | + |
| Midbrain tectum | N/A | N/A | N/A | | N/A | N/A | N/A | N/A | N/A | | N/A | N/A | N/A | N/A | N/A | | N/A | N/A | - | - | N/P | | - | + |
| Reticular formation | - | ++ | N/P | | - | N/P | - | - | N/P | | - | - | - | - | N/P | | - | - | - | - | N/P | | - | + |
| Substantia nigra pars compacta | + | ++ | N/P | | - | N/P | N/A | N/A | N/A | | N/A | N/A | N/A | N/A | N/A | | N/A | N/A | + | ++ | N/P | | - | + |
| Red nucleus | - | - | N/P | | - | N/P | N/A | N/A | N/A | | N/A | N/A | N/A | N/A | N/A | | N/A | N/A | N/A | N/A | N/A | | N/A | N/A |
| Locus coeruleus | N/A | N/A | N/A | | N/A | N/A | + | - | N/P | | - | + | - | +++ | N/P | | - | + | + | + | N/P | | - | + |
| Pontine tegmentum | - | ++ | N/P | | - | N/P | - | - | N/P | | - | + | - | ++ | N/P | | - | - | - | ++ | N/P | | - | - |
| Pontine base | - | ++ | N/P | | - | N/P | - | - | N/P | | - | - | - | +++ | N/P | | - | - | - | ++ | N/P | | - | - |
| XIIth nerve nuclei | N/A | N/A | N/A | | N/A | N/A | N/A | N/A | N/A | | N/A | N/A | N/A | N/A | N/A | | N/A | N/A | - | + | N/P | | - | - |
| Dorsal motor nuclei Xth | N/A | N/A | N/A | | N/A | N/A | N/A | N/A | N/A | | N/A | N/A | N/A | N/A | N/A | | N/A | N/A | - | - | N/P | | - | - |
| Inferior olive | N/A | N/A | N/A | | N/A | N/A | N/A | N/A | N/A | | N/A | N/A | N/A | N/A | N/A | | N/A | N/A | - | - | N/P | | - | - |
| Pyramid | N/A | N/A | N/A | | N/A | N/A | N/A | N/A | N/A | | N/A | N/A | N/A | N/A | N/A | | N/A | N/A | - | - | N/P | | - | - |
| Periaqueductal gray | N/A | N/A | N/A | | N/A | N/A | - | - | N/P | | - | - | N/A | N/A | N/A | | N/A | N/A | - | - | N/P | | - | + |
| Cerebellum | + | + | N/P | | N/P | - | + | + | N/P | | N/P | - | + | ++ | N/P | | N/P | - | + | ++ | N/P | | N/P | - |
| Vermis | ++ | ++ | N/P | | N/P | - | N/A | N/A | N/A | | N/A | N/A | N/A | N/A | N/A | | N/A | N/A | + | ++ | N/P | | N/P | - |
| Dentate nucleus | + | - | N/P | | N/P | - | - | - | N/P | | N/P | - | - | + | N/P | | N/P | - | - | + | N/P | | N/P | - |
| Key: - = absent; + = occasional/mild; ++ = moderate; +++ = severe/frequent, Tau [ - = absent; + = Neurofibrillary tangles/ abnormal neurites/neuropil threads; Aβ [D: diffuse plaques, M: mature plaques]; N/A: not available; N/P: not performed; Assessment: LB- α-syn, Tau- AT8, NL-H&E, Gliosis- GFAP; CAA- Aβ | | | | | | | | | | | | | | | | | | | | | | | | |

| **Additional file 1: Table S1: Semi-quantitative summary of neuronal loss (NL), gliosis pathology, Lewy bodies (LB), Tau pathology and Cerebral amyloid angiopathy (CAA) in DYT1 cases continued..** | | | | | | | | | | | | | | | | | | |
| --- | --- | --- | --- | --- | --- | --- | --- | --- | --- | --- | --- | --- | --- | --- | --- | --- | --- | --- |
| Brain regions | Case 5 | | | | | | Case 6 | | | | | | Case 7 | | | | | |
|  | NL | Gliosis | Aβ | | LB | Tau | NL | Gliosis | Aβ | | LB | Tau | NL | Gliosis | Aβ | | LB | Tau |
|  |  |  | D | M |  |  |  |  | D | M |  |  |  |  | D | M |  |  |
| Frontal cortex | - | + | + | ++ | - | + | - | + | ++ | + | - | + | - | ++ | N/P | | - | - |
| Temporal cortex | - | ++ | ++ | ++ | - | + | - | ++ | + | ++ | - | + | N/A | N/A | N/A | | N/A | N/A |
| Caudate | - | + | N/P | | - | + | - | ++ | N/P | | - | + | - | + | N/P | | N/P | - |
| Putamen | - | ++ | N/P | | - | - | N/A | N/A | N/A | | N/A | N/A | - | + | N/P | | N/P | - |
| Globus pallidus | - | ++ | N/P | | - | - | - | - | N/P | | - | - | - | ++ | N/P | | N/P | N/P |
| Thalamus | N/A | N/A | N/A | | N/A | N/A | - | + | N/P | | - |  | N/A | N/A | N/A | | N/A | N/A |
| Subthalamic nucleus | N/A | N/A | N/A | | N/A | N/A | - | ++ | N/P | | - | - | N/A | N/A | N/A | | N/A | N/A |
| Midbrain tegmentum | + | ++ | N/P | | + | - | N/A | N/A | N/A | | N/A | N/A | - | + | N/P | | - | N/P |
| Midbrain tectum | - | ++ | N/P | | - | + | N/A | N/A | N/A | | N/A | N/A | - | + | N/P | | - | N/P |
| Reticular formation | - | - | N/P | | - | - | N/A | N/A | N/A | | N/A | N/A | - | ++ | N/P | | - | - |
| Substantia nigra pars compacta | + | ++ | N/P | | ++ | + | - | ++ | N/P | | - | + | + | ++ | N/P | | + | - |
| Red nucleus | - | + | N/P | | - | - | - | + | N/P | | - | + | - | + | N/P | | - | N/P |
| Locus coeruleus | + | - | N/P | | ++ | - | N/A | N/A | N/A | | N/A | N/A | - | ++ | N/P | | - | + |
| Pontine tegmentum | - | - | N/P | | ++ | - | N/A | N/A | N/A | | N/A | N/A | - | ++ | N/P | | - | + |
| Pontine base | - | - | N/P | | - | - | N/A | N/A | N/A | | N/A | N/A | N/A | N/A | N/A | | N/A | N/A |
| XIIth nerve nuclei | N/A | N/A | N/A | | N/A | N/A | N/A | N/A | N/A | | N/A | N/A | N/A | N/A | N/A | | N/A | N/A |
| Dorsal motor nuclei Xth | N/A | N/A | N/A | | N/A | N/A | N/A | N/A | N/A | | N/A | N/A | N/A | N/A | N/A | | N/A | N/A |
| Inferior olive | N/A | N/A | N/A | | N/A | N/A | N/A | N/A | N/A | | N/A | N/A | N/A | N/A | N/A | | N/A | N/A |
| Pyramid | N/A | N/A | N/A | | N/A | N/A | N/A | N/A | N/A | | N/A | N/A | N/A | N/A | N/A | | N/A | N/A |
| Periaqueductal gray | N/A | N/A | N/A | | N/A | N/A | N/A | N/A | N/A | | N/A | N/A | N/A | N/A | N/A | | N/A | N/A |
| Cerebellum | ++ | ++ | N/P | | N/P | - | + | ++ | N/P | | N/P | N/P | + | ++ | N/P | | - | - |
| Vermis | + | ++ | N/P | | N/P | - | N/A | N/A | N/A | | N/A | N/A | + | ++ | N/A | | N/P | N/P |
| Dentate nucleus | - | - | N/P | | N/P | - | - | + | N/P | | N/P | - | - | - | N/P | | - | - |
| Key: - = absent; + = occasional/mild; ++ = moderate; +++ = severe/frequent, Tau [ - = absent; + = Neurofibrillary tangles/ abnormal neurites/neuropil threads; Aβ [D: diffuse plaques, M: mature plaques]; N/A: not available; N/P: not performed; Assessment: LB- α-syn, Tau- AT8, NL-H&E, Gliosis- GFAP; CAA- Aβ | | | | | | | | | | | | | | | | | | |
